# Contrary to endurance, power associated capacities differ between different aged and starting-nonstarting elite junior soccer players

**DOI:** 10.1371/journal.pone.0232118

**Published:** 2020-04-28

**Authors:** Matthias W. Hoppe, Vadim Barnics, Jürgen Freiwald, Christian Baumgart

**Affiliations:** 1 Institute of Movement and Training Science I, University of Leipzig, Leipzig, Germany; 2 Department of Movement and Training Science, University of Wuppertal, Wuppertal, Germany; eCampus University, ITALY

## Abstract

This study aimed to investigate differences in anthropometric characteristics and physical capacities (1) between under (U) 17, 19, and 21 years old elite junior soccer players, and also (2) between starting and nonstarting players within each age group. Ninety-two male elite German junior field players were tested for height, mass, fat, and fat-free mass as well as aerobic endurance, squat (SJ) and counter movement jump (CMJ), linear sprint, core strength-endurance, and one repetition maximum (1RM) bench press performance. According to their age and competitive match playing times, the players were divided into the mentioned different groups. Magnitude-based inferences and effect sizes (ES) were computed for statistical analyses. The fat-free mass, SJ and CMJ, 1RM bench press, and linear sprinting performances increased likely to most likely from U17 to U21 players (ES: moderate to large), whereas the body fat, core strength-endurance, and aerobic endurance performances remain constant. The fat-free mass, 1RM bench press, and linear sprinting performances were likely to most likely higher in U21 starting compared to nonstarting players (ES: moderate to large). Our study shows that contrary to endurance, power associated capacities differ between different aged and starting-nonstarting elite junior soccer players. This outcome should be considered for training, testing, and talent selection procedures in elite junior soccer players.

## Introduction

Soccer is an intermittent sport with periods of short high- and longer low-intensity actions [1]. Match play involves activities like running, jumping, dribbling, kicking, and tackling [2]; importantly, all within a technical-tactical context [1]. During competitive matches, professional players cover on average 9–14 km, whereas only 8–15% of that distance is considered to be high-intensity (≥19.8 km·h^-1^) [1]. The high-intensity actions last on average 2–3 seconds, occur every 60–90 seconds, and play a crucial role for defense and offensive play [3]. During the last decade, the high-intensity running distances increased up to ~35% in the English Premier League [4]. Due to this development, that can also be expected to have been occurred across all age groups [5], it is presently important to evaluate anthropometric characteristics and physical capacities of soccer players by specific testing procedures on a regular basis. Since it is well known that junior players perform more high-intensity actions during play than older players [6], a regular testing is especially important for juniors. This may help to optimize training drills and develop junior soccer players early in their career [7].

In soccer, it is accepted that anthropometric characteristics and physical capacities like muscle mass, speed, agility, repeated sprint ability, power, strength, maximum oxygen uptake, and intermittent endurance are important prerequisites to fulfill the playing demands [2, 8]. Thus, in soccer, knowledge of anthropometric characteristics and physical capacities is essential to optimize the training process on an individual basis [8], for example, according to the age of the players [9, 10]. In elite junior soccer players, it is known that the muscle mass [11, 12], repeated sprint ability [13], strength and power [14, 15], and speed [3, 10, 12, 14, 16, 17] capacities increase with maturation and training experience. Additionally, it is known that older elite junior soccer players have higher maximum oxygen uptakes than younger players [12, 15, 18]. Taken together, in soccer, the age of the players has an impact on anthropometric characteristics and physical capacities, which is important to consider for practical purposes as training, testing, and talent selection procedures.

Most of the published studies have compared anthropometric characteristics and physical capacities between under (U) 11 to 18 years old junior soccer players [12, 13, 15, 19, 20]. To our knowledge, there are only three studies that have investigated differences between older junior players [3, 12, 21]. Since the period from U17 to U21 is crucial for the players’ career to become a professional player [22], it is worth to examine differences in anthropometric characteristics and physical capacities between these age groups. Moreover, it is accepted that competitive matches induce the most specific, and thus effective training stimulus [23]. In this context, there are unfortunately only two studies that have compared anthropometric characteristics and physical capacities between starting and nonstarting elite junior soccer players, who differ in their competitive playing times [24, 25]. Thus, more research concerning differences in anthropometric characteristics and physical capacities between U17 to U21 years old elite junior, and also between starting and nonstarting, soccer players is needed.

This study aimed to investigate differences in anthropometric characteristics and physical capacities (1) between U17, U19, and U21 years old elite junior soccer players, and also (2) between starting and nonstarting players within each age group. It was hypothesized that there are differences in anthropometric characteristics and physical capacities between the different aged and starting-nonstarting players. Our findings may provide new practical relevant knowledge for training, testing, and talent selection procedures in the elite soccer environment.

## Materials and methods

### Participants and ethics statement

A total of 92 male elite junior field soccer players participated. The players competed in the German A/B-Junioren Bundesliga or in the 1^st^ to 4^th^ highest adult leagues. Twenty players played for the adult professional and 12 for their national teams. The players belonged to the most skilled juniors in Germany, trained on a professional daily basis, and had one competition per week during the season. All players were informed of the purposes, procedures, and potential risks of the study before they signed an informed consent document. Parental consents were given for the players younger than 18 years. All procedures were approved by the Ethics Committee of the University of Wuppertal (MS/JE 29.11.11) and were conducted in accordance with the Declaration of Helsinki.

### Experimental design

To investigate differences in anthropometric characteristics and physical capacities between the different aged and starting-nonstarting players, a retrospective study design over seven years (beginning 2012 to end 2018) was applied. The study was part of a comprehensive physical screening of soccer players from a total of 28 professional clubs. According to the competitions of the players, the testing procedures were performed at different points during the in-seasons. The tests were terminated on an individual basis in such a manner that the players had 48 to 72 h of recovery after their last competition. All players were instructed to perform no strenuous exercise the 24 h before testing and report well-hydrated to the tests. The tests were all standardized carried out at 9:00 in the morning.

Within a standardized test battery under laboratory conditions, anthropometric measures and the following physical performance tests in the mentioned order were performed: (i) a treadmill test, (ii) two vertical jump tests, (iii) a linear sprint test, (iv) four core strength-endurance tests, and (v) a bench press test. Between the tests, the players had 10 min of recovery, with exception after the treadmill test, whereby the players had 20 min. All testing procedures were conducted as described in detail elsewhere [26, 27].

According to their age and competitive match playing times, the players were divided into to the following groups: U17 (n = 38; starting = 19, nonstarting = 19), U19 (n = 31; starting = 18, nonstarting = 13), and U21 (n = 23; starting = 7, nonstarting = 16). To be considered as starting players, the players had to play: (i) >50% of all matches, (ii) >50% of playing time in each match, and (iii) be included for >50% in the starting eleven. The players, who were not able to fulfill these criteria, were considered as nonstarting players. The definitions based on previous research [24] and the required information were derived from the an internet portal [28].

### Data collection

To assess the body fat and fat-free mass, a 4-point bioelectric impedance analysis (Bodystat, QuadScan 4000, Douglas, United Kingdom) was performed in supine position, as described before [26]. The reliability of the bioelectric impedance analysis is ICC≥.90 [29].

To determine the aerobic endurance capacities, a ramp-like test on a motorized treadmill (H/P Cosmos Pulsar, Nussdorf-Traunstein, Germany) was conducted. Thereby, the following parameters were determined: time to exhaustion (Tlim), maximum oxygen uptake (VO2max), time to reach a respiratory exchange ratio of 1 (RER = 1), and running economy (RE) [30]. After a 4-min run at 10 km/h with 1% inclination, the latter was increased to 5% for further 4 min. Then, the speed was increased by 1 km/h every 2 min until maximal exhaustion was reached. The gas exchange was measured using an open-circuit breath-by-breath gas analyzer (Ganshorn, PowerCube-Ergo, Niederlauer, Germany) and averaged over 10 s throughout the test. The gas analyzer was calibrated with a calibration gas (15.5% O_2_, 5% CO_2_ in N; Messner, Switzerland) and a precision 1-l syringe (Ganshorn, Germany) according to the manufacture before each test. The Tlim was defined as the time from the start until the end of the test. The VO2max was defined as the highest value for oxygen uptake recorded during the test. The RER = 1 was defined as the time from the beginning until a RER of 1 was reached. The RE was calculated using the average oxygen uptake of the last 60 s at 10 km/h with 1% inclination. Before and immediately at the end of the test, capillary blood was sampled from the earlobe to determine the lactate concentration (EKF Biosen C_line Sport, Cardiff, United Kingdom). The achievement of VO2max was considered to be reached, if three of the following four criteria were met: (i) a plateau in oxygen uptake (increase <2 ml/kg/min) despite an increase in workload, (ii) a maximal respiratory exchange ratio >1.15, (iii) a maximal heart rate >95% of the age-predicted maximal heart rate (220-age), and (iv) a maximal lactate concentration >8 mmol/l [30]. The reliability of the aerobic endurance parameters is ICC≥.91 [31].

To assess the concentric and eccentric-concentric power capacities of the lower extremities, a squat (SJ) and counter movement jump (CMJ) test was performed. While the SJ test was conducted with the hands on the hips, the CMJ test was performed with an arm swing, as previously reported [26]. All jumps were performed on two separate force platforms that sample ground reaction forces at 1,000 Hz (Kistler, 9286BA, Winterthur, Switzerland). The vertical jump heights were calculated by the impulse-momentum method [32]. The players performed each jump test three times interrupted by a 2-min recovery period. The highest jumps were used for statistical analyses. The reliability of the SJ and CMJ tests is r≥.76 [33].

To assess the acceleration and sprinting capacities, a linear sprint test was conducted. The test was performed indoor on a plastic floor. The players started from a contact plate and sprinted over 30 m. The sprint times were recorded with double-light timing gates (TDS Werthner Sport Consulting, Linz, Austria) at 5, 10, 20, and 30 m. The players performed the test three times. Between each sprint, the players had 3-min for recovery. The fastest trail was used for statistical analyses. The reliability of the linear sprint test is ICC≥.73 [34].

To assess the core strength-endurance capacities of the ventral, lateral left and right, and dorsal core muscles, four separated tests were performed. The tests consisted of repeated concentric-eccentric exercises over defined movement ranges, as described in detail elsewhere [26]. For all tests, a movement frequency of 1 Hz was dictated by a metronome (KDM-1, Korg, Inagi, Japan) and time to exhaustion was measured using a stopwatch. The sum of all tests was used for statistical analyses, as previously conducted [26]. Between each core test, the players had a 5-min recovery. The reliability of the strength-endurance tests is r≥.80 [35].

Finally, to assess the upper body strength capacities, a bench press test was performed. The test was performed with a 20-kg competition style bar (Gym80, International Sygnum Basic, Gelsenkirchen, Germany). After a standardized warm-up, the weights were increased in 5-kg increments until a one repetition maximum (1RM) was reached. A trial was considered to be successful, when the players had lowered the bar downwards to their chest without bouncing and pushed the bar upwards until their arms were fully extended [27]. Between each trial, the players had a 3-min break. The reliability of the bench press test is ICC≥.91 [36].

### Statistical analysis

Unfortunately, our retrospective study design did not allow to generate comparable sample sizes across the different groups and also not to perform a sample size calculation prior to the player recruitment. Consequently, to investigate differences in means, Magnitude-based inferences that are independent of the sample size, and thus are well suited for unique low sample sized populations [37] were computed, as described in detail elsewhere [38]. Briefly, means, 90% confidence intervals (CI), and percentage differences were calculated first. Then, the disposition of the CI for all differences in relation to the smallest worthwhile difference (SWD) were examined. The SWD was defined as the pooled standard deviation multiplied by 0.2, as previously conducted [38]. The likelihoods for the differences “truly” being higher, similar, or lower than the SWD were determined and qualitatively described using the following probabilistic scale: <1%, most unlikely; 1 to <5%, very unlikely; 5 to <25%, unlikely; 25 to <75%, possibly; 75 to <95%, likely; 95 to <99%, very likely; and ≥99%, most likely. If the likelihoods for having both higher and lower values were 5%, the differences were described as unclear. Otherwise, the differences were interpreted according to the observed likelihoods, whereas only those differences rated as at least likely (>75%) were considered [39]. Finally, standardized differences reported as effect sizes (ES) were calculated and interpreted as: trivial (<0.2), small (0.2 to <0.6), moderate (0.6 to <1.2), large (1.2 to <2.0), very large (2.0 to <4.0), and extremely large (≥4.0) [38].

## Results

Table 1 summarizes the descriptive data for all assessed variables.

**Table 1:** Descriptive data for all assessed variables. The data are means±90% confidence intervals.

Variable	U17			U19			U21		
	All (n = 38)	ST (n = 19)	NST (n = 19)	All (n = 31)	ST (n = 18)	NST (n = 13)	All (n = 23)	S (n = 7)	NST (n = 16)
**Age (years)**	15.6±0.2	15.8±0.2	15.5±0.3	17.6±0.2	17.4±0.2	18.0±0.4	19.9±0.3	20.0±0.6	19.8±0.3
**Height (m)**	1.78±0.01	1.79±0.02	1.77±0.02	1.79±0.01	1.80±0.02	1.77±0.02	1.80±0.02	1.81±0.03	1.79±0.03
**Mass (kg)**	68.2±1.8	70.0±1.9	66.4±3.0	72.6±1.7	73.3±2.7	71.6±1.6	77.7±1.7	79.6±3.4	76.8±1.9
**Fat (%)**	9.8±0.8	9.8±1.2	9.8±1.1	10.1±0.8	10.1±1.3	10.0±1.0	10.8±0.9	9.6±1.1	11.3±1.1
**FFM (kg)**	61.6±1.8	63.2±2.1	60.0±2.9	65.2±1.5	65.7±2.2	64.4±1.7	69.3±1.7	72.0±3.6	68.1±1.8
**Tlim (s)**	966±27	986±37	935±37	988±33	984±40	970±56	953±25	939±70	961±21
**VO2max (ml/kg/min)**	56.9±1.5	55.8±1.9	56.4±2.4	54.9±1.3	55.3±1.6	53.6±2.3	55.0±1.7	54.8±2.4	55.1±2.3
**RER = 1 (s)**	643±46	636±67	638±64	586±52	588±82	581±50	611±57	606±86	616±75
**RE (ml/kg/min)**	36.7±1.6	35.2±1.1	38.1±2.9	33.5±1.3	34.1±1.8	32.8±1.9	34.3±1.4	36.3±1.1	33.2±1.8
**1RM BP (kg)**	64±3	65±4	62±4	74±3	73±4	75±3	82±4	89±7	79±4
**SJ (cm)**	33.5±1.0	33.8±1.6	33.1±1.3	35.5±1.2	35.1±1.5	35.6±1.9	38.2±2.3	38.9±3.4	37.9±3.0
**CMJ (cm)**	40.3±1.3	40.5±1.8	40.0±1.8	41.6±1.2	42.1±1.6	41.0±2.1	45.2±2.5	47.4±4.1	44.2±3.1
**Total core (s)**	354±20	352±27	355±30	339±27	365±36	304±36	348±33	368±37	338±52
**5 m (s)**	1.109±0.008	1.101±0.011	1.116±0.015	1.090±0.012	1.087±0.019	1.094±0.014	1.087±0.014	1.050±0.025	1.104±0.016
**10 m (s)**	1.847±0.013	1.840±0.015	1.854±0.023	1.822±0.015	1.826±0.027	1.817±0.018	1.808±0.021	1.762±0.037	1.828±0.021
**20 m (s)**	3.130±0.021	3.108±0.026	3.151±0.034	3.086±0.024	3.096±0.039	3.073±0.023	3.038±0.033	2.969±0.056	3.067±0.037
**30 m (s)**	4.326±0.032	4.287±0.042	4.365±0.053	4.265±0.035	4.280±0.058	4.244±0.032	4.181±0.048	4.089±0.081	4.222±0.058

U17, U19, and U23 = Under 17, 19, and 23 years old players; n = Sample size; ST = Starting players; NST = Nonstarting players; FFM = Fat-free mass; Tlim = Time to exhaustion; VO2max = Maximum oxygen uptake; RER = Respiratory exchange ratio; RE = Running economy; 1RM = One repetition maximum; BP = Bench press; SJ = Squat jump; CMJ = Counter movement jump.

### Different aged players

**Fig 1:**
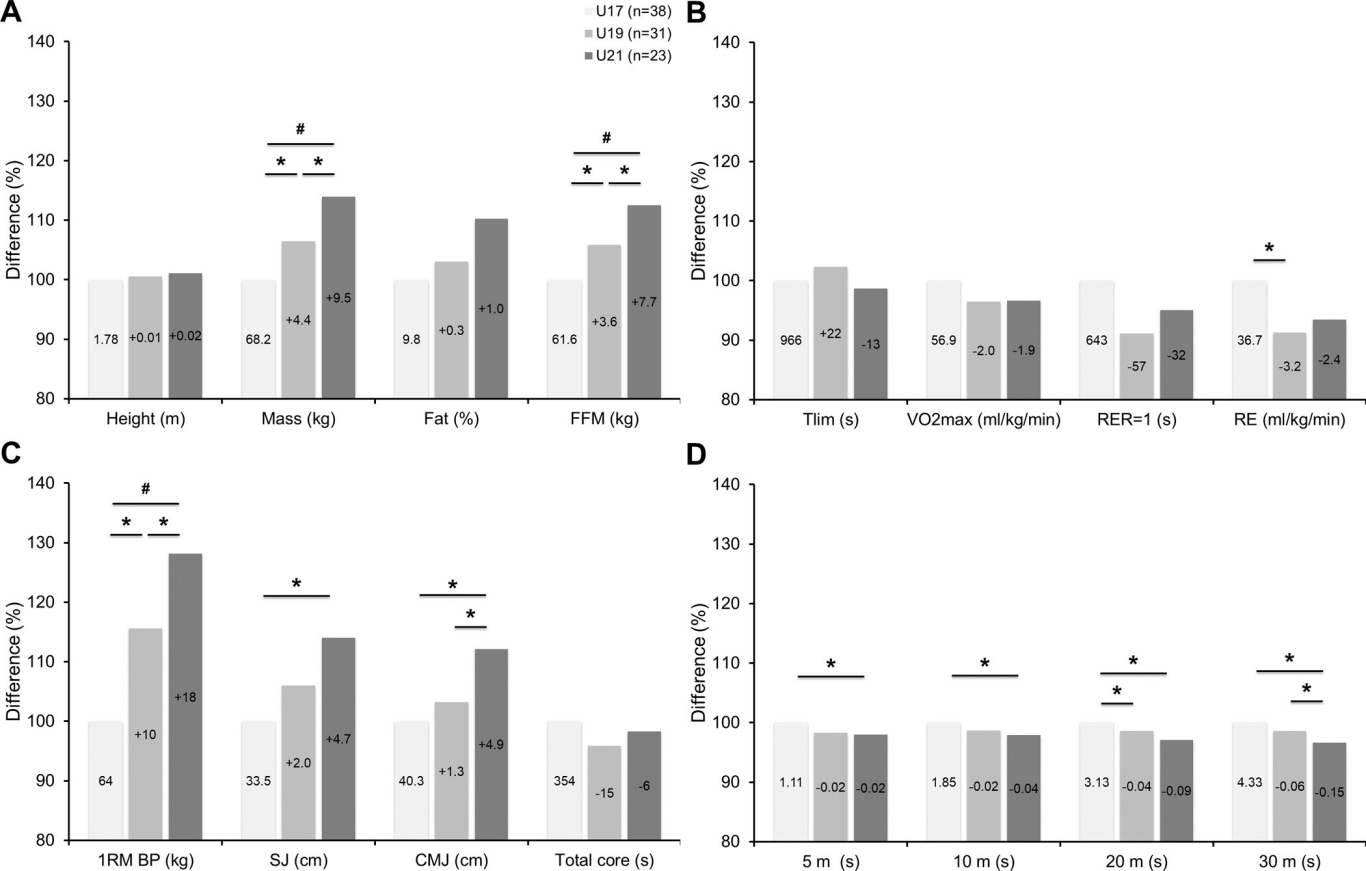
Differences in anthropometric characteristics (A) and physical capacities (B-D) between U17, U19, and U21 years old elite junior soccer players. Bars show relative differences in relation to U17 players. Within bars, absolute data and differences in relation to U17 players are also shown. U17, U19, and U21 = Under 17, 19, and 21 years old players; n = Sample size; FFM = Fat-free mass; Tlim = Time to exhaustion; VO2max = Maximum oxygen uptake; RER = Respiratory exchange ratio; RE = Running economy; 1RM = One repetition maximum; BP = Bench press; SJ = Squat jump; CMJ = Counter movement jump; * = Moderate effect size; # = Large effect size.

Fig 1 shows the differences in anthropometric characteristics (A) and physical capacities (B-D) between the different aged players. The U21 players have a most likely higher mass (ES: moderate to large) and fat-free mass (ES: moderate to large) than U19 and U17 players. The U19 players have a very likely higher mass and fat-free mass (ES: moderate) than U17 players. The U19 players have a very likely lower RE (ES: moderate) than U17 players. The U21 players have a very likely to most likely superior 1RM bench press performance (ES: moderate to large) than U19 and U17 players, most likely superior SJ performance (ES: moderate) than U17 players, and likely to very likely superior CMJ performance (ES: moderate) than U19 and U17 players. The U19 players have a most likely superior 1RM bench press performance (ES: moderate) than U17 players. The U21 players have a likely superior 30 m linear sprinting performance (ES: moderate) than U19 players and likely to most likely superior 5, 10, 20 and 30 m linear sprinting performance (ES: moderate) than U17 players. The U19 players have a likely superior 20 m linear sprinting performance (ES: moderate) than U17 players.

### Starting and nonstarting players

**Fig 2:**
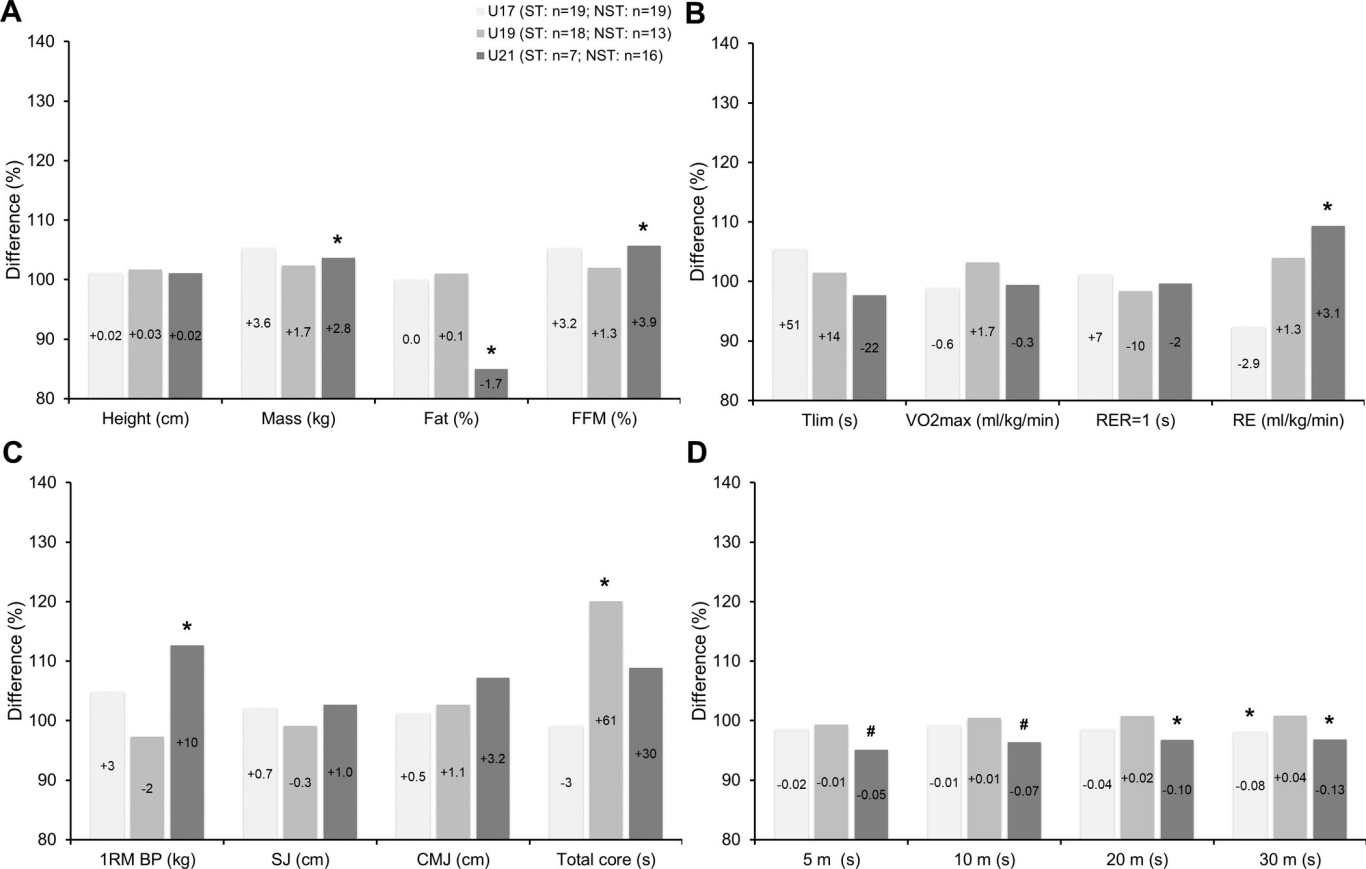
Differences in anthropometric characteristics (A) and physical capacities (B-D) between starting and nonstarting elite junior soccer players within U17, U19, and U21 age groups. Bars show relative differences in relation to starting players. Within bars, absolute differences in relation to starting players are also shown. U17, U19, and U21 = Under 17, 19 and 21 years old players; n = Sample size; ST = Starting players; NST = Nonstarting players; FFM = Fat-free mass; Tlim = Time to exhaustion; VO2max = Maximum oxygen uptake; RER = Respiratory exchange ratio; RE = Running economy; 1RM = One repetition maximum; BP = Bench press; SJ = Squat jump; CMJ = Counter movement jump; * = Moderate effect size; # = Large effect size.

Fig 2 shows the differences in anthropometric characteristics (A) and physical capacities (B-D) between the starting and nonstarting players within the different age groups. The U21 starting players have a likely higher mass, likely lower body fat, and likely higher fat-free mass (ES: moderate) as well as likely worse RE (ES: moderate), very likely superior 1RM bench press performance (ES: moderate), and very to most likely superior 5, 10, 20, and 30 m linear sprinting performance (ES: moderate) than U21 nonstarting players. The U19 starting players have a likely superior total core performance (ES: moderate) than U19 nonstarting players. The U17 starting players have a likely superior 30 m linear sprinting performance (ES: moderate) than U17 nonstarting players.

## Discussion

This study aimed to investigate differences in anthropometric characteristics and physical capacities (1) between U17, U19, and U21 years old elite junior soccer players, and also (2) between starting and nonstarting players within each age group. It was hypothesized that there are differences in anthropometric characteristics and physical capacities between the different aged and starting-nonstarting players. Our main findings were: (1) The fat-free mass, vertical jump height, 1RM bench press, and linear sprinting performances increased from U17 to U21 players, whereas the body fat, core strength-endurance, and aerobic endurance performances remain constant. (2) The fat-free mass, 1RM bench press, and linear sprinting performances were higher in U21 starting compared to those of the U21 nonstarting players.

Considering our first main finding (Fig 1), the identified age related differences between U17, U19, and U21 elite junior soccer players are in line with those of previous studies that have investigated differences in fat and fat-free mass [11, 12], aerobic endurance [18, 21], vertical jump height [12, 14, 15, 17], and linear sprinting [3, 14–17] performances. One explanation for our found increased fat-free mass, vertical jump height, 1RM bench press, and linear sprinting performances may be that the muscle mass and its neuronal control improve during the naturally occurring growth, development, and maturation processes, for example, due to increased testosterone and growth hormone levels and neuronal myelination, respectively [40]. Beside these naturally occurring processes, a further possibility may be that there also occur neuromuscular adaptions, as a consequence of the systematic soccer training [7, 19] and participation in competitions [25, 41]. More research to clarify both assumptions is needed. Thereby, in elite junior soccer players, it is important to control for the interindividual variation in the maturity status [9], for example, by peak height velocity measures [42]. Since biological and chronological ages can differ up to four years [43], the different maturation processes may have had an impact on our measures; especially, in our younger age groups.

Noteworthy, the found lack of increased aerobic endurance and core strength-endurance performances question their construct validities for elite junior soccer players. In this context, it is worth mentioning that the aerobic endurance performance has not changed in Spanish [44] and Norwegian [21] elite players over the last 10 years, and does also not determine the professional career of elite junior soccer players [44]. Contrary, it has been shown that sprints and jumps precedes 67% of all goals during match play, and thus play a crucial role within decisive situations in elite soccer [45]. Therefore, it is reasonable that professional coaches place highest priorities on power capacities in elite soccer players [3, 21, 46]. With respect to the core strength-endurance, it has been shown that our applied tests do not replicate soccer specific movements and are not related to strength and power [47], but rather to aerobic endurance capacities [27]. Further studies to examine the construct validity of both endurance related capacities for junior elite soccer players are warranted.

Regarding our second main outcome (Fig 2), the revealed differences between starting and nonstarting U21 elite junior soccer players are also supported by the findings of the few previous studies [24, 25]. The previous studies show that U19 starting players have superior linear sprinting, agility, and vertical jump performances than nonstarting players at the end of the season [25]. Other research with professional players from the English Premier League shows that starting players perform more high-intensity actions than nonstarting players during all training sessions and competitive matches across a season [48]. Also, research has shown that starting English Premier League soccer players have improved vertical jump and peak power performances three days after match play. This suggests that match play can be considered as an effective training stimulus to improve power capacities of professional players [41]. Moreover, it is well known that power and speed are the most important physical capacities in elite soccer players [3], which may all support our outcomes. The reasons may be due to the player selection by the coaches for the competitions and again to gained adaptions. It can be that more powerful players are predominately nominated by the coaches for the competitions at an elite junior soccer level [24]. Thus, U21 elite junior soccer players with higher power capacities may likely benefit to be selected to compete on an elite level. Otherwise, players with more match playing time may benefit from adaptions inducted by competitions [25, 41]. Since all of our recruited players trained on a professional daily basis, our found differences between starting and nonstarting player are likely related to their different match playing times. Worth mentioning is, however, that also the sprinting and jumping performances do not determine the promotion from elite junior to professional soccer players [49].

While our study increases the knowledge in the elite soccer environment, few limitations have to be acknowledged. First, our retrospective study design did not allow mechanistic discussions and strong conclusions, whether the found differences are related to growth, developmental, and maturation or training and competition induced adaptations for which (controlled) longitudinal studies are needed [50]. Second, our test battery was completed at different time points during the in-seasons. The reason was that our test battery was a part of a comprehensive physical soccer player screening. Therefore, we investigated junior players not from a single, but rather from numerous professional clubs, which can also be seen as a methodological strength concerning the generalization of our findings. Third, during our test battery, we performed a treadmill test until exhaustion first, which may had induced fatigue and affected the following test results. However, we performed the treadmill test first to provide a standardized warm-up procedure in a laboratory setting and gave the players 20 min to recover, which can be considered to be sufficient on an elite level [13]. Additionally, it has to be considered that of the ~15 min lasting treadmill test, only ~5 min were spent at high-intensity with a RER>1 (Table 1). Last, the impact of our findings on the match play performances remained unknown. Consequently, further research to address these points is required.

## Conclusions

Our study shows that contrary to endurance, power associated capacities differ between different aged and starting-nonstarting elite junior soccer players. This outcome should be considered for training, testing, and talent selection procedures in elite junior soccer players. With this in mind, our findings indicate that power capacities should be optimized in U17, U19, and U21 years old elite junior soccer players. These capacities can be addressed by soccer-specific resistance, plyometric, or sprint drills [51]. Since our findings also question to place a focus on aerobic training, it is suggested to integrate–if required–the aerobic training into the technical-tactical training [52] or to perform small sided games [53–55]. Moreover, our outcomes suggest that coaches should replicate the competitive playing demands by specific training drills or friendly matches to provide a sufficient training stimulus for the nonstarting players. Concerning testing procedures, our findings propose that it is presently more important to evaluate power than endurance capacities in elite junior soccer players. Lastly, our results may be useful for talent selection and development processes. Elite junior soccer players with superior developed power capacities may likely benefit to be selected for competitions. Consequently, the development of power capacities should take place at an early stage of the career.

## Supporting information

S1 Raw dataset (XLSM)
